# The New York Institute of Stomatology

**Published:** 1899-07

**Authors:** 

**Affiliations:** The New York Institute of Stomatology


### Reports of Society Meetings.

### THE NEW YORK INSTITUTE OF STOMATOLOGY.

A regular meeting of the Institute was held on Tuesday evening, April 4, 1899, at the office of Dr. C. O. Kimball, 27 West Thirty-eighth Street, the President, Dr. E. A. Bogue, in the chair.

The minutes of the last meeting were read and approved.

### SPECIAL BUSINESS.

*Dr. R. H. M. Dawbarn.*—On account of the very interesting features of this case, especially in connection with your specialty of our profession, I have induced this lady to be present this evening. The affection was diagnosticated by her physician, Dr. Freudenthal, as sarcoma of the antrum; later she was presented at the New York Academy of Medicine, where this diagnosis was confirmed by a number of surgeons present, and the question was there discussed as to what should best be done. The case was finally sent to me, I being especially interested in this field. The symptoms have only been noticed for about two months. Within a week or two after they were first observed the growth increased so rapidly that it involved surrounding parts, and now the nasal passage on the right side is absolutely air-tight. It has lately encroached on the orbit, from beneath, so as to cause a distinct occurrence of exophthalmos; indeed, it will require an extremely delicate operation to save the eye, and if this were to be postponed many days, I do not think the eye could be saved.

A point of especial interest is that, at the beginning of the symptoms, the neuralgic pains led her to go to her dentist, who extracted five teeth of the upper arch; three of these were from the right side and two from the left. I can hardly see how any reputable dentist could perform such an operation. I think this is a supreme instance of an opportunity thrown away by a dentist (and an opportunity that so many dentists do throw away) in not looking beyond the teeth and their condition, thus neglecting to do their patient the greatest service by calling attention to abnormalities at a time when surgical interference is attended with so much better results than is the case with malignant growths a little later. Dr. Howe, who is with us to-night, will remember an instance in which he took advantage of this opportunity. He had the wisdom to look beyond the teeth, and made a brilliant diagnosis of what proved to be a malignant growth; this was three years ago. I excised the upper jaw in that case, and, as he knows, the patient is still well and free from any recurrence.

To demonstrate the process of bone-softening, which the bony tissue over the antrum has undergone in this case, I will ask the president to pass an ordinary sewing needle through this growth into the antrum. He will find that, instead of there being the densest of dense bone which is found normally over the antrum, he will be able to pass the needle through with very little sense of resistance.

*The President.*—I find, on passing the needle as directed by Dr. Dawbarn, a condition producing a tactile sensation, very much like a dry sponge. Like cartilage rather than bone in its resistance to the needle thrust.

*Dr. Dawbarn.*—On Friday this lady will consent, as the only means of saving her life from this form of cancer, to an excision of the entire superior maxilla, part of the nasal bone, a portion of the lachrymal bone, most of the malar bone, about one-half of the palate bone, and the entire inferior turbinated bone.

The periosteum forming the floor of the orbit will, if apparently sound, be retained. If the case had been seen a little earlier, it is probable that the bony walls and floor of the orbit could have been left intact, and the moderate degree of diplopia, which will now be evident, could have been avoided. The incision will run along the naso-facial junction, splitting the middle of the lip, and following those lines which will produce as little apparent change in the contour of the face as possible.

The external carotids must be tied to begin with, in order that the operation be less perilously bloody.

The lady has asked me if this meant that her face would be spoiled, and I have assured her that, thanks to the successful work of the gentlemen in your line of our profession, this need not be the case. The amount of deformity will probably be very little, indeed. In Dr. Howe's case the prosthetic appliance, made by Dr. Bishop, was excellent. The patient has been enabled to continue the practice of his profession,—the law,—and I do not think any one, at a distance of three feet, would detect any disfigurement; also his articulation of sounds is entirely clear. At a later period I shall ask some member of this society for something in the way of a plumper for the cheek, and a half-set of upper teeth to help out in the appearance of this patient.

*Dr. George S. Allan.*—Are there any symptoms, and if so, what symptoms might we look for as indicating the presence or approach of sarcoma?

*Dr. Dawbarn.*—There are absolutely none, except a lump, a prominence, a swelling. There is not always softening of the bone present, although it is a rather early sign, as a rule. The wise plan is to excise a small piece of any suspicious growth, under cocaine, and have it examined by a competent pathologist.

*Dr. Allan.*—Would a dentist be warranted in doing this?

*Dr. Dawbarn.*—Perhaps not. He should refer the case to some surgeon. Any swelling is abnormal, and it is the province and duty of the dentist, in making his examination of the teeth, to note any such condition about the mouth, and call immediate attention to it, provided that he does not think that the state of the teeth can account for the tumefaction.

*Dr. J. F. P. Hodson.*—Were there, in your estimation, any dental predisposing causes?

*Dr. Dawbarn.*—Not in the least, in this case; there was, however, pain referred to the teeth, and undoubtedly this is why the dentist extracted five of them. I believe such a practitioner is a disgrace to his profession. I have always regarded even a partially decayed tooth as simply a good friend in distress, and worthy of all care and attention.

*Dr. Hodson.*—Is it probable that this growth could have occurred, even in the antrum, without some irritating cause?

*Dr. Dawbarn.*—We do not know as yet the causes from which spring malignant growths, either sarcomata or carcinomata. At the latest meeting of the New York State Medical Society, held at Albany, and at which I was present as a delegate, this question was taken up by a pathologist working in the laboratory of the University of Buffalo. He gave certain reasons for believing that malignant growths were micro-organic in their nature. We have long suspected this, but it has never been proven with absolute certainty. It seems quite probable that, in view of the recent work in this direction, the State Society will receive a subsidy from the Legislature with which to pursue further investigations.

Because of the great and steady increase in frequency of cancerous affections all over the world, which is now well known to be the case, such a subsidy would seem a wise investment of public money.

*Dr. J. Morgan Howe.*—Is there any characteristic pain usually associated with malignancy?

*Dr. Dawbarn.*—I know of none. These tumors, like every other tumor, after they have grown to a certain size, begin to encroach upon the surrounding parts, thus irritating the sensory nerves by pressure, producing the same degree of pain as do other tumors in the same situation. I know of no suffering diagnostic and peculiar to these growths.

I would say that if any member of the society is interested, I would feel very much honored to have him present at the operation, which will take place at the Polyclinic Hospital on Friday of this week, at my clinic.

### COMMUNICATIONS ON THEORY AND PRACTICE.

*Dr. C. O. Kimball.*—I have the pleasure of presenting a paper by Dr. L. C. Bryan, of Basel, Switzerland, entitled, "Securing Loose Teeth affected with Pyorrhœa." I will pass around a model exhibiting a case with Dr. Bryan's appliance in position.

(For Dr. Bryan's paper, see page 436.)

*Dr. G. H. Maxfield.*—I will pass around a cast of the lower arch of a patient who came under my care two months ago for treatment of pyorrhœa. The cast is of interest as showing a case of bridge-work which I found in the mouth. I wish to call attention to the size of the gold cap which formed one of the abutments of the bridge as compared with the size of the natural first bicuspid.

*Dr. L. C. Leroy.*—The two models, an upper and a lower, which I will pass around are from a case in which an ill-fitting plate that had been worn for some time has caused hypertrophy of the lip.

*The President.*—We will now have the pleasure of hearing the paper of the evening, entitled "Pyorrhœa Alveolaris from a Bacteriological Stand-Point," by Dr. W. J. Younger, of Chicago.

(For Dr. Younger's paper, see page 413.)

DISCUSSION.

*Dr. E. C. Briggs.*—I have been greatly interested in the new ideas advanced by Dr. Younger, but I regret that I am unable to discuss this paper from the stand-point of a bacteriologist, inasmuch as this is, to me, a new presentation of the subject. I have had some experience with this affection, however, from a clinical stand-point which may be of interest, and which I shall be very glad to relate. I have felt that pyorrhœa alveolaris was not essentially due to micro-organisms, but that, after the production of the pocket or recesses, there was an invasion by the common bacteria of the mouth and the pus-forming bacteria, the process going on, if not checked, to the loss of the teeth thus affected. I also hold that this condition is largely controlled by constitutional conditions; that individuals of the sthenic type yield much more readily to treatment than those of the asthenic.

In uric acid diathesis, complications arise which require treatment for the relief of the constitutional conditions. However, aside from the possible constitutional treatment, which any given case may call for, this disease requires the most vigorous local treatment. Regarding the importance of this local treatment, I most heartily agree with Dr. Younger; indeed, it was Dr. Younger who first impressed me with the importance of a thorough mechanical cleansing of these pus-pockets.

It would seem from his recent investigations that the object of this operation is not only to remove the calculi, the detritus, and pyogenic bacteria, but for the removal of a special bacterium identified with this disease. However that may be, I cannot emphasize too much the importance of a thorough cleansing of the teeth affected with pyorrhœa alveolaris. So essential is this for a permanent cure that I do not hesitate, in many cases, where I find it impossible to perfectly cleanse a tooth, to extract such a tooth, and, after carefully removing the deposit on the roots, replace it. I find this is attended with a most admirable and successful recovery.

The fixation of the teeth during the treatment I find of great importance, and, apropos of Dr. Bryan's paper, I have frequently supported the lower teeth by Dr. Draper's method, which consists of striking up a splint to go back of the teeth, with pins running clear through the upper third of the incisors. These pins are cut with a screw thread, and, when the appliance is set in cement, everything is made rigid by screwing nuts on these pins. These nuts are afterwards removed.

After the mechanical treatment, I believe in the thorough destruction of the pyogenic membrane and of any diseased soft tissue which may be in the pocket. For this purpose I use a mixture of caustic potash and carbolic acid, or a solution of chloride of zinc. I also use lactic acid, trichloracetic acid, and aromatic sulphuric acid, not with any expectation that these substances will soften or dissolve any deposit of tartar which might be present.

I also wish to lay great stress upon the treatment which the patient is able to give himself at home, treatment of an antiseptic and stimulating nature. A stimulating treatment is especially essential in patients of the asthenic type, where the system is sluggish and there is a great lack of tone. In these cases massage is attended with marked beneficial results. Bicarbonate of soda is useful, the patient applying it himself, depositing it about the teeth at frequent intervals. I have frequently placed around these teeth, while under treatment, loose bands of pure silver, with very satisfactory results, probably on account of the formation of silver salts by the action of the buccal fluids. It has the advantage over the lactate or nitrate of silver in that there is less discoloration.

Regarding the cause of the disease, it does not seem to me that pyorrhœa alveolaris is very different in its nature, or in its cause, from the trouble which so often occurs around a partially erupted wisdom-tooth. In these cases where the crown of the tooth is just peeping through the gum we have a condition most favorable to the lodgement of *débris* from the mouth and bacteria between the gum tissue and the enamel, there setting up an irritation followed by a discharge of pus very similar to conditions about a tooth affected with pyorrhœa.

There is one other thing I wish to mention in this connection,—the value of the pulp in a fully developed tooth. As I understand it, the pulp is a constructive organ, and I believe from my experience that its usefulness is practically at an end when the tooth is fully made, and after that period its presence becomes a greater source of trouble and danger than a cause of peace and happiness. The removal of the pulp by means of arsenous acid or its death by approach of decay I do not consider a scientific removal, but if the pulp can be removed surgically and the canal immediately filled without the parts becoming infected, I feel that that tooth is just as good as it was before and just as vital in its associations and connections in the mouth, and by so doing I have prevented any trouble which may come from an irritation of that pulp, especially in pyorrhœa alveolaris, and also prevented the formation of pulp-stones and exostosis. The practice of taking out teeth for the purpose of thoroughly cleansing them in the treatment of pyorrhœa reveals a great many diseases due to irritation of the pulp. A not infrequent cause of trouble is the death of the pulp in one of the roots of a multirooted tooth, a condition which would be very difficult to diagnosticate. For these reasons, one of the first things I do for a tooth seriously affected with pyorrhœa is to surgically remove the pulp and fill the canals.

*Dr. S. E. Davenport.*—I hope Dr. Briggs will be willing to say a few words or more regarding the extraction and replacement of teeth in the treatment of pyorrhœa alveolaris. Will he kindly inform us whether he prepares the sockets in any particular manner, and also if this procedure applies to both single and multirooted teeth.

*Dr. Briggs.*—It was Dr. Younger's operation of transplantation which first led me to feel that these teeth could be treated in this way. The process holds good for both single- and double-rooted teeth. After extracting the tooth I inject the socket with a weak solution of cocaine, and drill it as deep as it seems advisable in that particular location. I find in this disease that the tissues at the apex are not in the best condition, and I deem it advisable to deepen the socket to some extent beyond this point.

I have noticed in the transplantation of teeth, in one instance in particular, that by considerably deepening the socket, a firmer union is produced. In this case I had measured the length of the tooth preparatory to drilling the socket, and in drilling the socket overlooked the fact that I had measured the entire length of the tooth instead of only the root. I think I never had a case which behaved better than this in every way. The tooth became like a rock, and has since been wedged in order that something might be done to an adjoining tooth.

*The President.*—Will Dr. Briggs kindly inform us how he removes a pulp surgically, when that pulp happens to be in a lower molar; the lower molars having two roots and frequently four canals with a web between?

*Dr. Briggs.*—In cases where I have extracted the tooth, if unsuccessful in removing the pulp from the opening in the crown, I fill from the apex of the root, consequently whatever organic matter may remain is sealed in the canal and can give no trouble. I extract pulps from teeth in the mouth, with twenty-per-cent. solution of cocaine, by the method described in the INTERNATIONAL DENTAL JOURNAL by me some years ago.

*Dr. S. A. Hopkins.*—I would like to ask Dr. Younger to tell us a little more about his methods of procedure, which led him to the conclusion that this particular form of bacillus was the cause of pyorrhœa alveolaris. As I understand, there were eight cases, and cover glasses as well as cultures were made from each of these cases, the bacillus being found on the cover-glass preparation as well. Were other mouths examined to ascertain if the bacillus was present in healthy mouths as well?

*Dr. Younger.*—No examinations were made from healthy mouths.

*Dr. Hopkins.*—The disease was not produced by inoculation?

*Dr. Younger.*—Only in the rabbit.

*Dr. Hopkins.*—But the particular disease of pyorrhœa alveolaris has not been produced by inoculation?

*Dr. Younger.*—This we could not expect without inoculation of human beings, an arrangement for doing which is now under consideration.

*Dr. Hopkins.*—Galippe described an organism more than ten years ago, which he thought was the specific organism of pyorrhœa alveolaris, and which, according to his description, was very similar to the one described by Dr. Younger. He described it as being at first almost like a coccus, then growing out into long rods, frequently giving almost a leptothrix appearance. May I ask Dr.

Younger if the inoculations made with the organism growing on the surface of the serum or with the liquefied serum contained the bacillus? In the latter case the effects produced on the animals inoculated might have been due to the toxic effect of the serum, and not to the presence of the bacteria. In my examinations of these cases I have made many cover-glass preparations, and in most cases have found the staphylococcus pyogenes aureus and sometimes other pus-forming bacteria. I have not found anything that would produce pyorrhœa when inoculated. Allow me to say, with all due deference, that I think the case cannot be considered as proven until the disease is produced by inoculation in a dog, or some susceptible animal, or in a human subject.

*Dr. Younger.*—I do not ask that this micro-organism be accepted absolutely as the specific germ of pyorrhœa alveolaris at the present time, as we have not carried our investigations far enough as yet to make definite statements. I have only given the results of the investigations thus far, and, as I have said, will probably be able in a short time to report the effect of inoculations in human beings.

*Dr. Allan.*—The dog is susceptible.

*Dr. Younger.*—We cannot always judge accurately of the action of a bacterium on a human subject by its effect on a lower animal. For this reason I shall endeavor to get a human subject; failing that, I shall fall back on the dog.

I wish to say that in the preparation of these cultures the greatest care has been used, and precautions even greater than described in my paper have been observed.

Regarding the bacterium described by Galippe, as I remember, the micro-organism, which he described as a "delicate, double-bulbed parasite, changing into a bacillus by culture," was found in the tubuli of the dentine, and not in the canaliculi of the cementum. Besides, I cannot understand how a parasite can change into a bacillus.

*Dr. Gordon White.*—It has been my privilege to have been in Dr. Younger's office a number of times, and to have seen him operate in the manner which he has described to us to-night. I have never made any bacteriological examinations of these conditions, but in recent years I have pursued rather closely Dr. Younger's methods, and to him, perhaps, I am indebted more than to any other individual for my success in the treatment of pyorrhœa alveolaris. My first serious attention was called to the disease some sixteen years ago, when I found its presence in my mother's mouth. This case I treated with aromatic sulphuric acid, which I believe was first recommended by Dr. Atkinson. I have had occasion to treat this case four times since then, with excellent results. To-day I am treating these cases, with more or less success, by Dr. Younger's method, with this exception. I have fancied, from ill-effects in several cases, that the lactic acid was liable to undergo a fermentation, and for this reason I add to the acid, about four drops to the ounce, formalin.

The formaldehyde also seems to have the effect of hardening the gums more rapidly, and in my hands it has been, I think, an excellent adjunct to lactic acid. I believe it generally conceded that formaldehyde is one of our most powerful antiseptics, and will prevent decomposition of flesh indefinitely, as it is claimed a piece of beef immersed in a solution of say a tablespoonful of formalin to a barrel of water is placed in a thoroughly aseptic condition, its surface is hardened and putrefaction prevented. It may have some such effect when placed in the pockets caused by pyorrhœa alveolaris.

I have been very much interested in the subject of the evening, and wish to thank Dr. Younger for his excellent paper.

*Dr. Hodson.*—On account of the absence of Dr. Stewart I have been asked by Dr. Kimball to relate to the Institute the results of Dr. Stewart's clinic before the Odontological Society on the treatment of pyorrhœa alveolaris. As a matter of fact, I am almost as ignorant of the minutiæ of what was done at that clinic as those who were not there. Inasmuch as I am conductor of the clinics, I feel it incumbent upon me, as host, to make sure that every one else sees the operations, and so see very little of them myself.

The theory, however, upon which Dr. Stewart works is this: He believes pyorrhœa alveolaris to be chiefly due to over-calcification of the tooth itself, and especially of the cementum covering its roots, and more or less hypertrophy of the latter as well. His treatment consists, first, in a very careful, thorough, and sure removal of all the calcific deposit, either salivary or serumal, and the removal at the same time of at least one-third of the thickness of the cementum covering the root, as far down as he can get at it.

The treatment thereafter is with pure hydrochloric acid, for the purpose of decalcifying the surface of the cementum. Instead of the usual striving to produce as little inflammation as possible, he, on the other hand, endeavors to produce as much as possible. In addition to the acid treatment and the removal of the deposit and surface of the cementum he makes three or four grooves lengthwise of the root, in the belief that the new growth dipping into these grooves will make a firmer attachment by this process of ankylosis, or whatever the new bony attachment may be. At any rate, the results are excellent.

The process is preceded by free injection of eucaine into the gum, this in preference to cocaine, because the former produces no constitutional symptoms or after-effects.

The treatment is, as I said, very successful, so much so as is indicated by the fact that we have seen cases of the disease, both before and after Dr. Stewart's treatment, which others had failed to cure, and the most it had seemed possible to do was to hold the disease in control by constant treatment, which cases, when treated by Dr. Stewart, resulted in an apparently perfect and positive cure. I do not know but that the same result may have been produced by Dr. Younger's method, but this method has accomplished a cure of pyorrhœa alveolaris that I have never seen equalled.

*Dr. W. G. A. Bonwill.*—As much as I respect Dr. Younger, I am really surprised at his article to-night. It seems to me that it does not at all agree with his former practice and teachings.

It was during his practice in San Francisco that he taught us that the treatment of this disease was almost entirely mechanical. In his paper to-night he would have us believe that the cause of pyorrhœa alveolaris is a special bacillus at the apex of the root. For forty-six years I have been in practice, and in all those years I have yet to see the first case of pyorrhœa develop in my practice. Patients over whom we have control, and whose teeth we treat from the beginning and who come to us at frequent intervals and have their teeth cleansed, never develop this disease. Three-quarters of the cases, I believe, come from improper methods of filling teeth, without observing the contour of the tooth as it originally was, and leaving faulty approximal spaces into which food can be crowded. Malocclusion is also a very powerful cause, and it is my invariable practice, after having thoroughly cleansed the teeth, to take impressions of the mouth and study the articulation. Ninety per cent. of the cases can be cured by looking to these details. After thoroughly cleaning the teeth and taking your impressions leave the pockets alone. There is nothing so sure to keep up the trouble as frequently poking instruments into these places.

I am perfectly willing to take my chances against this disease where I have control over my patients from the beginning, and as for bacteria, I have very little regard for them.

*Dr. J. Morgan Howe.*—I have been greatly pleased with Dr. Younger's paper, which I consider one of the best presented papers—in the sense of a clear presentation of the author's meaning—I have ever listened to at a dental meeting. I will not attempt to discuss it, but I wish to emphasize what the essayist has stated, as to the value and necessity of removing all the deposit from the roots of teeth affected with pyorrhœa.

To accomplish this in cases where it has been otherwise impossible, I have for some time past followed the method spoken of by Dr. Briggs, of extracting the teeth, and, after cleansing and filling the roots, replanting them. This has been attended with quite a respectable percentage of successes.

The removal of the deposit is a prerequisite to complete success, and should not be overlooked or neglected.

*Dr. S. E. Davenport.*—I wish to make my acknowledgments to Dr. Younger for favoring us this evening, and also for the service his special instruments have rendered my patients during the past few years in the treatment of pyorrhœa alveolaris. We all know how thoroughly Dr. Younger goes into everything he undertakes, and how successful he has been in other directions; we feel, therefore, like accepting most heartily the practical points at least of his conclusions, concerning pyorrhœa alveolaris and its treatment.

We are also greatly indebted to Dr. Briggs for coming to us this evening, and I believe I have derived great benefit from what has been said. There is one point in Dr. Briggs's remarks which I feel ought not to pass without something further being said on the subject. I understood him to say that he considered the pulp to be of no value to the fully developed tooth; that its only function was that concerned in the development, and that after this period he had no hesitancy in extirpating the pulp. I hope I misunderstood him, and I believe that the majority of those present will differ from this view. In my opinion, the living pulp is of value to the adult tooth, if for no other reason than for the additional strength and elasticity which it imparts.

It is quite possible, however, that Dr. Briggs refers only to those teeth so affected with pyorrhœa alveolaris that there is a tendency to disturbances in the pulp, in which case I should find no fault with its surgical extraction. My mind goes back to a meeting of a dental society last year, where a New York practitioner made the sweeping assertion that the pulp was of no value to a tooth after it had arrived at its full growth, and a gentleman here to-night, to his honor be it said, took the opposite view and very ably sustained his position.

*Dr. Briggs.*—I am very glad that Dr. Davenport has mentioned this subject again. Of course, I would not undertake to remove the pulp from a perfectly sound tooth. The removal only comes in connection with the treatment of disease, but I still think that while the presence of a living pulp may have certain advantages in the way of resiliency and elasticity, perhaps, these advantages are greatly over-balanced by the disadvantages, for with the pulp comes pulp-stones, exostosis, and death of the pulp.

*Dr. A. H. Brockway.*—In other words, the value of the pulp diminishes with the maturity of the tooth. I quite agree with this.

*Dr. Hopkins.*—I would like to ask Dr. Kimball if he recalls, in Dr. Dunning's practice, a case of pyorrhœa alveolaris in one of Dr. Dunning's patients, who had been constantly under his care, and who appeared for examination and treatment whenever he wished him to do so; whether it is not true that Dr. Dunning eliminated pyorrhœa alveolaris from his practice?

*Dr. C. O. Kimball.*—My own personal experience, running back thirty years, is very much on a line with that of Dr. Bonwill and that of many other gentlemen present. So far as I can speak of Dr. Dunning's practice, a certain small percentage of which has come into my hands, it would seem that in those patients whose mouths were closely watched and the tartar carefully and thoroughly removed I cannot recall a single case of pyorrhœa alveolaris. Of course, there may be cases which have drifted beyond my knowledge. I have seen one or two cases in my own practice which have puzzled me, cases which have not responded to treatment. In the case of molar teeth it has sometimes seemed impossible to thoroughly remove the deposit at the bifurcation of the roots. I suppose if I had more boldness in operation I should adopt Dr. Briggs's method of extracting the tooth. However, that has not been my plan. I can only say that I heartily corroborate Dr. Bonwill's views, that in cases which have been carefully watched and the tartar thoroughly removed pyorrhœa alveolaris does not occur.

*The President.*—It seems to me that we have touched upon a subject this evening which interests us very much. The last speaker voices the experience of a great many of the older practitioners present. He might even go farther, and assert that, with the exception of a few cases of systemic disease, such as gout, rheumatism, and diabetes, pyorrhœa alveolaris does not exist where the teeth have been properly cared for.

I was pleased that Dr. Davenport asked Dr. Briggs the question he did, for I have felt that Dr. Briggs should tell us more regarding this subject, and also regarding his method of surgically extirpating the pulps of teeth; and I want to put a large interrogation-point after this, for I believe there are many teeth in the mouth, especially molars and some bicuspids, the roots of which cannot be surgically cleared by the hand of man. I feel quite sure that no gentleman present feels himself with Voltaire "above his Creator in knowledge." All good and useful teeth should have pulps, and unless they have been subjected to great neglect at some stage of their existence, they will not need to have their pulps extirpated.

To return to the treatment of pyorrhœa, which has been so ably dwelt upon, Dr. Younger mentioned two or three things on which I should be obliged if he would still further enlighten us. Among other things, he stated that a large percentage of the human animal —about ninety per cent.—was more or less afflicted with this disease. He also stated that he did care to experiment on dogs in the matter of inoculation for fear of inaccuracy. If the injection of a pure culture is to be made upon a human subject, I would suggest it be upon one of the ten-per-cent. immunes. Dr. Bonwill brought out a truth which is not very often called to our attention. He was very careful to emphasize the fact that the articulation had much to do with the presence or absence of tartar. The extraction of one or more teeth from the normal thirty-two allows more or less tipping or displacement of the remaining teeth, leaving spaces and protected nooks favorable to the deposition of tartar. To give a very personal illustration of this: I have lost just two teeth, a lower molar and an upper bicuspid, extracted in the hope of correcting an irregularity existing in the incisors. After twenty-five years I found a space existing between a canine and bicuspid, the breadth of a finger-nail, with a collection of tartar in this situation. I forced these teeth together, closing up the spaces, and have held them closed, and since then have had no trouble with the collection of tartar. I have seen this in a great many cases, and only allude to it, as I do not see it generally mentioned in our literature.

*Dr. Bonwill.*—There is one other thing I wish to speak of in connection with articulation in these cases. It is the desirability of restoring, by removable sections, any teeth which may have been lost, thus bringing back the normal occlusion, making two perfect arches. I prefer removable pieces which can be easily cleaned. A bridge in these situations is very undesirable, as it cannot be properly cleansed, and is very apt to result in the production of pyorrhœa. A proper occlusion is so important that it is the first step in the treatment of the disease, and not only should we look for the restoration of lost parts, but extruded teeth should be ground and the articulation so corrected that each tooth does its share, and only its share, of the work to be done. All fillings should be contoured to the original form of the tooth. It is this malocclusion which is the cause of more cases of pyorrhœa than any other one thing.

*Dr. Dawbarn.*—It seems to me that the essayist has put his finger, without question, upon the cause of this disease. I am not a specialist in this line, but as a specialist in another line of our common profession, I should be greatly surprised if there were not some microbic cause for this affection.

To be sure, I think there are undoubtedly two factors,—the mechanical irritation of the tartar and the microbic irritation.

We all know that when we have such mechanical irritation it distinctly tends to the formation of pus with very much less microbic infection than otherwise would be the case.

Mechanical irritation by the formation of tartar undoubtedly is a predisposing cause, but the exciting cause must be microbic, unless we have here the anomaly of all scientific surgery. Whether the individual microbe which is being studied to-night is the right one is another question, and one which will never be settled definitely until the disease has been produced by inoculation of a human subject. I have experimented in other fields upon dogs and other lower animals, and could mention many instances where dogs differ strikingly from the human being. For instance, as to the action of anæsthetics, when the famous experiments were carried out through the Nizam of Hyderabad, by the English Chloroform Commission experimenting on dogs, it was found that these died always from respiratory failure. This we know is not always the case in the human being. Dogs also react differently to heart-stimulants, and there is also a marked irregularity of rhythm in the beating of a dog's heart; no dog ever has a regular pulse. Another point in which they differ is in their response to morphine-poisoning. Some little time ago it was stated by Dr. Moor, of New York, that he could cure morphine-poisoning by the administration of permanganate of potassium; that he had experimented by injecting several grains in a dog, and afterwards exhibiting successfully the antidote. The fact is that it is difficult to kill a dog with morphine. They can receive three, four, or five grains hypodermically without death ensuing. For these and other reasons results cannot be considered absolutely conclusive until the disease has been produced by inoculation of a human being; though tests on the lower animals are useful and, indeed, indispensable.

*Dr. Younger.*—I regret that I have a very poor memory, and trust I may be excused if I fail to answer all the questions which have been asked. As I have said, I do not claim that it is absolutely certain, beyond a doubt, that I have discovered the bacterium of pyorrhœa. I only stated that the results thus far obtained point very strongly in this direction. In seven cases out of eight we have succeeded in finding a bacterium which has not been before described. Regarding Dr. Bonwill's statement, that these theories do not agree with my former experience and teaching, I fail to see upon what ground he bases this opinion. Undoubtedly malarticulation is a cause; but I do not give this factor the importance that Dr. Bonwill does. I believe that pyorrhœa is the cause of malocclusion much more often than is malocclusion the cause of pyorrhœa. In the papers which I have had the honor of having Dr. Bonwill listen to, one in San Francisco and the other in Moscow, I gave the same mechanical and traumatic reasons as the causes that bring about pyorrhœa that I give in this paper, and which I will mention more fully later on. At that time I had in mind these bacterial theories, but was not ready to announce them, and said nothing about the microbic side of the question, except in my Moscow paper, in which I promulgated the theory that the source of the deposit was from the alveolar substance; that, by an inflammatory action the walls of the alveolar process were broken down, its lime salts precipitated or liberated in minute division, and taken up by the pus or exudate, and were by force of impact deposited on the root (the pericementum having already been removed by inflammatory destruction), and agglutinated there by bacterial energy; that the deposit upon the root is usually in proportion to the amount of wasting of the alveolus, and *vice versa.* There is nothing in my former statements which conflicts with my paper tonight. But in this paper I had thought to deal more minutely with the bacterial side of the question, and I only regret that I did not have two or three more weeks in which to perform some experiments upon human individuals.

In its inception, the disease is not bacterial, but traumatic. An inflammatory action has first to be established which will reach to the alveolar process. This is effected by the lodgement in the fold of the gum that surrounds the cervix of the tooth of some insoluble substance, as little grains of sand, seeds of berries, husks of oatmeal, minute flakes floating in beer, little fragments of the skin of fruit or their core, bristles from the tooth-brush, food detritus, etc., which, by persistent presence, awakens irritation, then inflammation and destruction of the soft tissues, until the peridental membrane is reached, involved, and punctured, and the alveolar process exposed. It is at this time that the nutrient material is presented that invites the pyorrhœal bacterium to establish its virulent energies. It is not until this is accomplished that pyorrhœa alveolaris commences. Whatever experiments we make, whether in the lower animals or the genus homo, I believe it will be necessary for success that this inflammatory condition, reaching to the alveolar process, be established before placing the microbe. I think the puncture of the peridental membrane must always precede the invasion of the microbe. I feel certain that if the microbes are injected into healthy gum, or where the vitality of the tissue has not been sufficiently lowered, that the flow of serum induced by the lesion would be sufficient to destroy their vitality, or to at least render them inert. And this is the reason that the experiments of Miller and others in the mouth of dogs—presuming for argument's sake that they had my bacillus—were fruitless. It is this fact—this having to prepare a nutrient condition for the bacterium beforehand—that will, I think, make it difficult to establish all of Koch's requisites to prove a specific bacterium for this disease.

Of course, there is a great deal in the power of resistance in the individual, but it is only in the progress of the disease that this power is manifested, because, in a lowered condition of systemic vitality, the proteid molecules are more easily broken down or disintegrated by bacterial action. Perfect systemic health will not immune, no more than a vitiated constitutional condition will produce pyorrhœa.

*Dr. F. Milton Smith.*—I have been very much interested in Dr. Younger's remarks, and was also pleased to hear Drs. Bonwill and

Kimball say that cases of pyorrhœa alveolaris seldom have developed in their practice where they have had control of their patients from early life. It does not seem to me that there is anything antagonistic between the grounds taken by Dr. Younger and these gentlemen. The thought has occurred to me many times that, if we could have entire control of our patients, keeping the gums in a healthy condition, and the teeth cleansed, we would have no difficulty in keeping our patients free from the disease; but if such is not the case and the gums become unhealthy and detached from the necks of teeth, it would seem that then we have a favorable condition for these bacteria to set up the trouble. I am very glad that Dr. Younger has treated the subject in a practical way, for, as another has said, it is a condition that confronts us and not a theory. It is not so much a question of how we shall treat those whom we know are safe, but what we shall do for the many who are already in trouble. I move that we extend a vote of thanks to Dr. Younger for his excellent paper, and to Drs. Briggs, Hopkins. and Bonwill for their assistance in the discussion.

Carried.

Adjourned.

FRED. L. BOGUE, M.D., D.D.S.,
*Editor The New York Institute of Stomatology.*

